# Supplementary material for: Prostate Cancer Cells Are Sensitive to Lysosomotropic Agent Siramesine through Generation Reactive Oxygen Species and in Combination with Tyrosine Kinase Inhibitors
Source: Cancers (Basel). 2022 Nov 8;14(22):5478. doi: 10.3390/cancers14225478 (PMC9688505; doi:10.3390/cancers14225478)
Supplement: Supplementary file 1 [file cancers-14-05478-s001.zip › cancers-1969400-supplementary.pdf]

Supplemental Figure S1

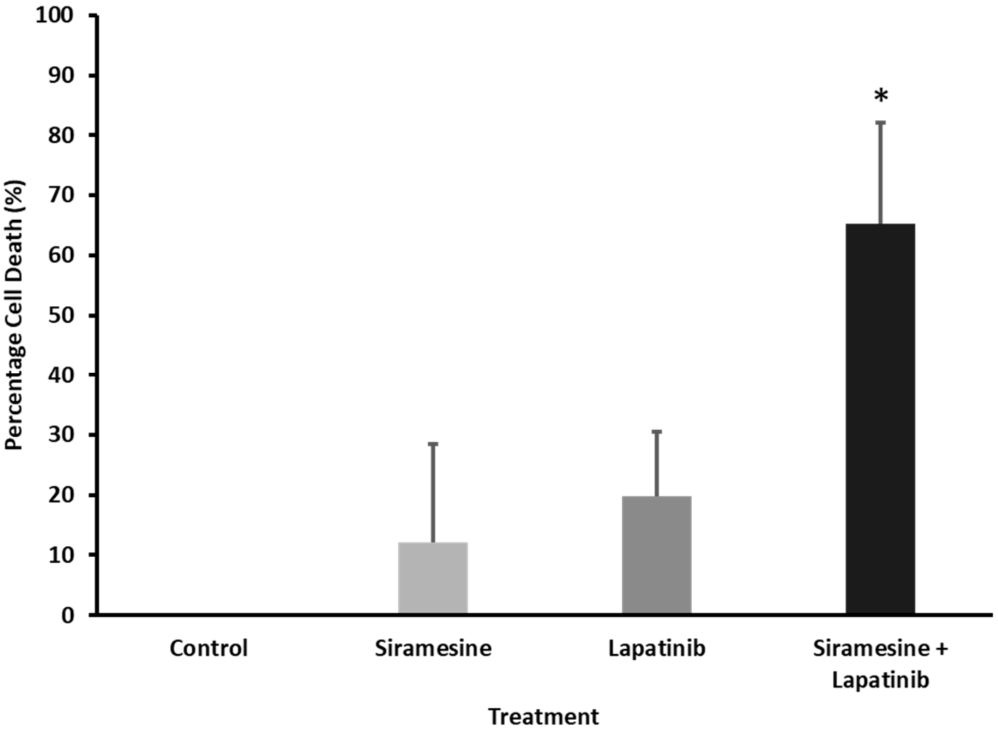

Supplementary Figure S1. Siramesine and lapatinib increased cell death. A MTS cell viability assay performed on 1.5×10^4 PC3 cells seeded in each well of a 96-well plate treated with DMSO as a negative control, 0.5 µM lapatinib, 10 µM siramesine, or a combination of siramesine and lapatinib for 24 hours. Percentage cell death was normalized to negative control. $p<0.05$*, $p<0.01$**, $p< 0.001$***, $p< 0.0001$****. n=3.

Supplemental Figure S2

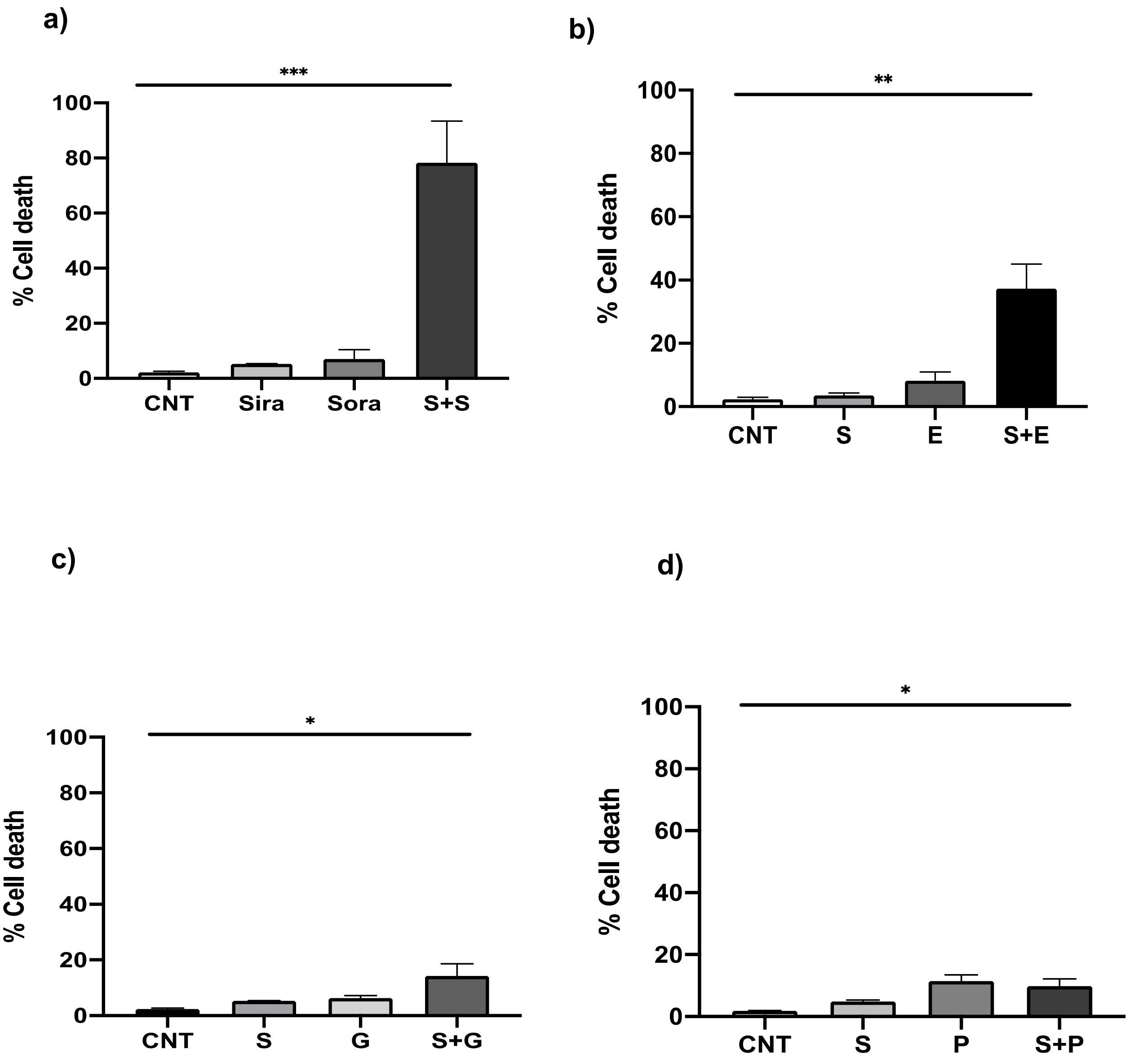

PC3 cells

Supplemental Figure S2. Siramesine and sorafenib induce synergistic cell death in PC3 cells. PC3 cells were treated with siramesine (Sira, 10 uM) and sorafenib (Sora 10μM) (a) or siramesine (S, 10μM) and gefitinib (G, 10μM) (b) for 24h and stained with the fluorescent dye Trypan blue (0.4%) and analyzed by flow cytometry. An increase in fluorescence indicates an increase in cell death. Results are representative of at least three independent replicates (N=3).PC3 cells were treated with siramesine (Sira, 10 μM) and etoposide (E, 200 μM) (a) or siramesine (S, 10 μM) and paclitaxel (P, 1 μM) (b) for 24h and stained with the fluorescent dye Trypan blue (0.4%) and analyzed by flow cytometry. An increase in fluorescence indicates an increase in cell death. Results are representative of at least three independent replicates (N=3).

Supplemental Figure S3

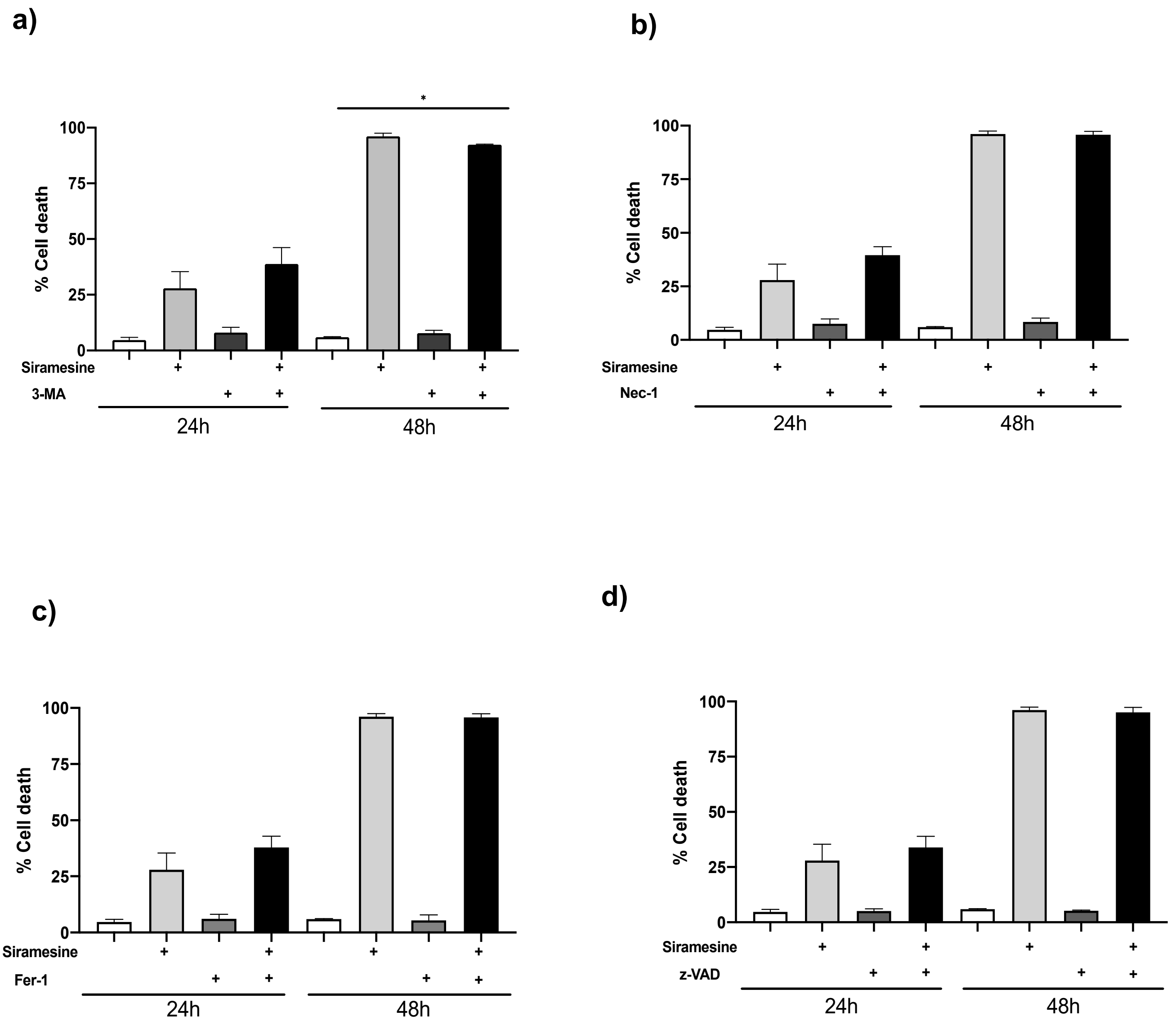

PC3 cells

Supplemental Figure S3. Inhibitors of autophagy, necroptosis, ferroptosis and apoptosis fail to block cell death after treatment with siramesine. PC3 cells were treated with siramesine (20µM) for 24h and 48h. Before treatment, cells were incubated with either 3-MA (2 mM) (a), Nec-1 (10µM) (b), Fer-1 (5µM) (c) and z-VAD (10µM) (d) for 1h at 37C. Cell death was quantified by Trypan blue (0.4%) and analyzed by flow cytometry. Results are representative of three independent replicates (N=3).

Supplemental Figure S4

A)

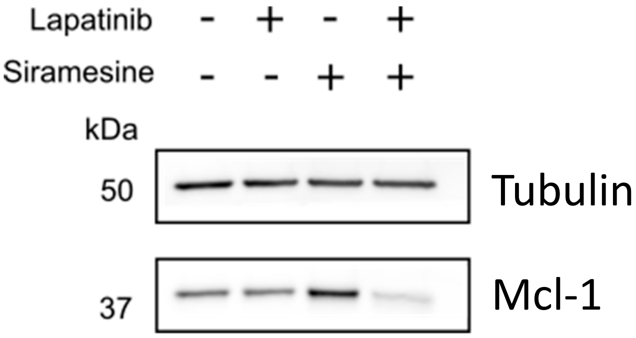

B)

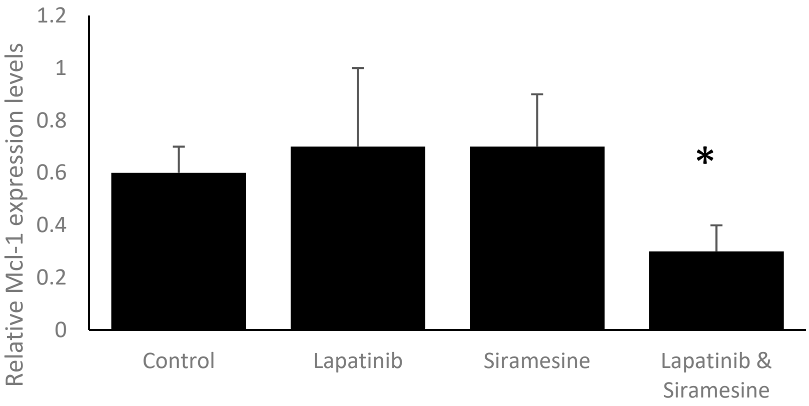

Supplemental Figure S4. Siraminse and lapatinib treatment decrease expression of Mcl-1 PC3 cells were treated with siramesine (2.5µM) and/or lapatinib (0.5µM) for 24h. The cells were lysed and western blotted for Mcl-1 and reprobed for tubulin as a loading control. This result represents three independent experiments.

Supplemental Figure S5

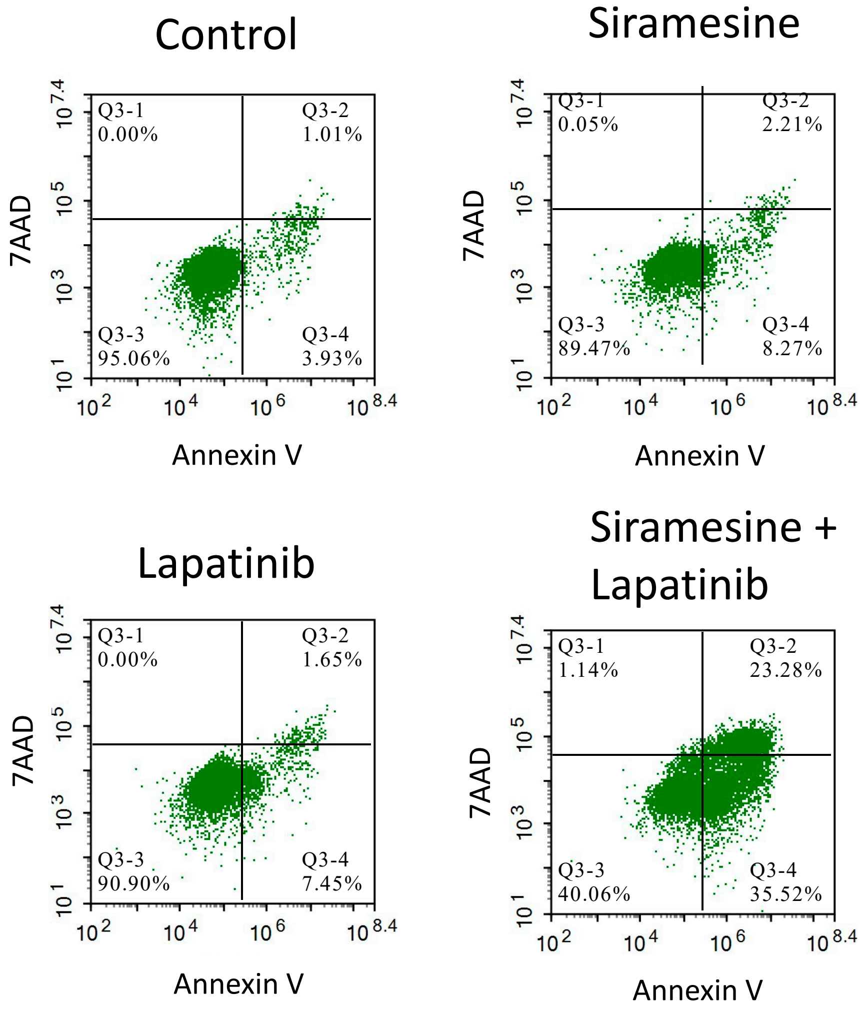

Supplemental Figure S5. Siramesine and lapatinib induce apoptotic cell death
PC3 cells were untreated, treated with siramesine alone (S, 10mM), lapatinib alone (L, 0.5 mM) or in combination. (a) After 24 hours, cells were incubated with AnnexinV/7AAD dyes for 15 minutes and analyzed by flow cytometry. Results are representative of three independent replicates (N=3).

Supplemental Figure S6

A)

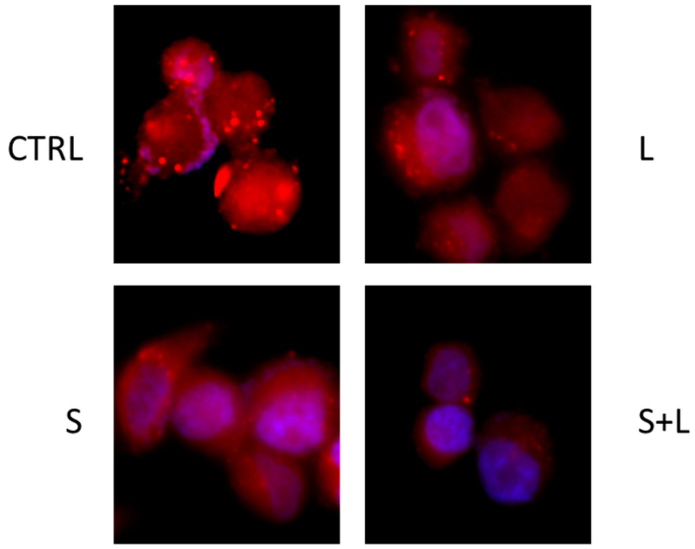

B)

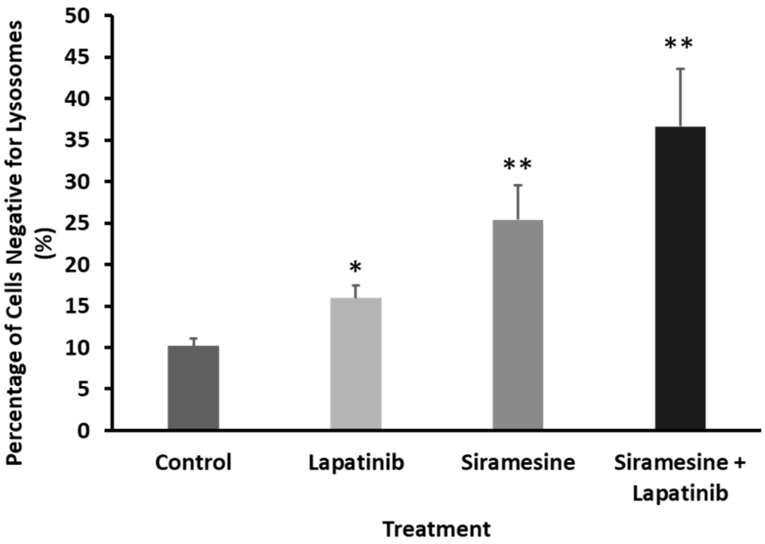

Supplemental Figure S6. Siramesine and lapatinib partially induce lysosomal membrane permeabilization (LMP) at 4h. PC3 cells were treated with with DMSO as a negative control, 0.5 µM lapatinib, 10 µM siramesine or a combination of siramesine and lapatinib for 4h. After treatment, cells were stained with Lysotracker red (50 nM) and DAPI for 15 minutes at 37°C and LMP was visualized by fluorescence microscopy. Loss of red fluorescence indicates lysosome disruption. Results are representative of three independent experiments (n=3) and 200 cell counts per replicate. p<0.05*, p<0.01**.

Supplemental Figure S7

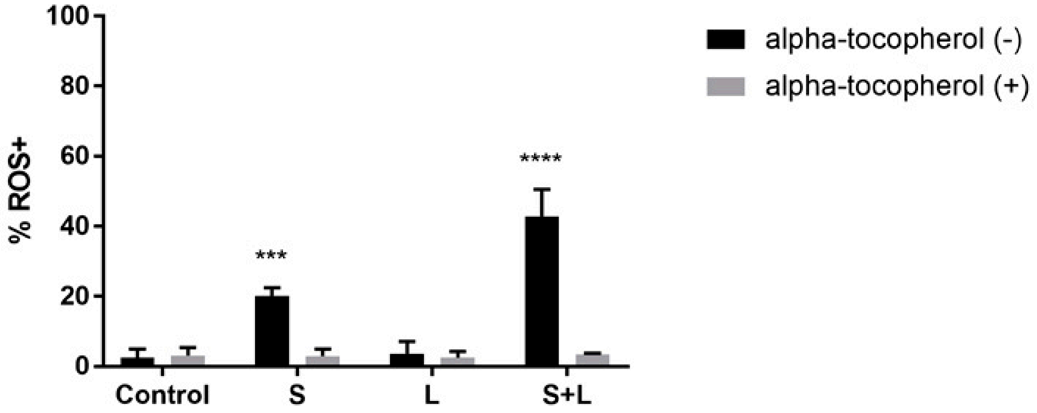

Supplemental Figure S7. Alpha-tocopherol reduces ROS levels following siramesine and lapatinib treatment. PC3 cells were treated with 10µM siramesine and 0.5mM lapatinib for 24h. To detect reactive oxygen species, cells were stained with 3.2mM DHE for 30 minutes at 37C. An increase in fluorescence indicates an increase in reactive oxygen species as determined by change in percentage of ROS positive cells (% ROS+). All these experiments were analyzed by flow cytometry. Results are representative of three independent replicates (N=3) with error bars representing standard error. *** represents significant difference of p <0.005
